# Mononucleosis: A Possible Cause of Idiopathic Hypersomnia

**DOI:** 10.3389/fneur.2018.00922

**Published:** 2018-10-31

**Authors:** Emilia Sforza, David Hupin, Frédéric Roche

**Affiliations:** Service de Physiologie Clinique et de l'Exercice (Pole Hospitalier NOL), CHU Nord, Faculté de Médecine Jacques Lisfranc, Université Jean Monnet, SNA EPIS EA 4607, Université de Lyon, Saint-Étienne, France

**Keywords:** Idiopathic hypersomnia, mononucleosis, inflammation, immunological processes, exessive daytime sleepiness

## Abstract

Idiopathic hypersomnia (IH) is a rare central hypersomnia of unknown physiopathology. In this study, we determine if the presence of infectious mononucleosis evaluated by serological markers of Epstein Barr virus infection plays a role in this hypersomnia. Ten patients with a suspicion of IH underwent to clinical assessment, 24 h polysomnography, and serologic testing for mononucleosis including Viral Capside Antigen (VCA) IgG, the VCA IgM, and the EBV nuclear antigen (EBNA). None of the patients reported neurological inflammatory disease and viral infection prior the onset of the disease. Compared to the laboratory serological reference values, all patients had high levels of VCA IgG and EBNA with lower level of VCA IgM, overall indicating past infection. This study shows that prior infectious mononucleosis may predispose some subjects to idiopathic hypersomnia suggesting the role of inflammatory and immunological processes in this sleep disorder.

## Introduction

Idiopathic hypersomnia (IH) affects more frequently young adults and comprise a combination of symptoms characterized by daytime sleepiness and a decreases amount of alertness during daytime despite long sleep time lasting more than 10 h (1, 2). Associated symptoms are long and un-refreshing naps, difficulty waking up, sleep drunkenness in the morning, sometimes depressive and anxiety symptoms, and a tendency to sleep phase delay (3). The diagnosis of IH relies on the exclusion of other cases of hypersomnia secondary to other sleep disorders, i.e., narcolepsy without cataplexy, sleep related breathing disorder, periodic legs syndrome, sleep deprivation, irregular sleep schedule and/or to head trauma, medication use, substance abuse or neurological diseases such as myotonic dystrophy and multiple sclerosis (4).

Although the clinical spectrum of IH is to date well defined, the pathogenesis remains largely unknown. A previous study (5) examining the published cases of symptomatic narcolepsy and HI conclude that despite the lack of hypocretin ligand deficiency in the symptomatic cases the hypothesis of hypothalamic lesions involving the orexin system may be hypothesized. Moreover, since histamine is one of most important neurotransmitter promoting wakefulness, in animal (6, 7) and humans (8), a study (9) in patients with narcolepsy, idiopathic hypersomnia or obstructive sleep apnea shown reduced cerebrospinal fluid histamine levels only in narcolepsy and HI cases suggesting the hypothesis of a lowering of histamine levels in central hypersomnia. In a paper published by Guilleminault and Mondini (10), the authors reported the presence of daytime sleepiness in 10 patients suspected to have Epstein-Barr viral infection in some cases with immunological neurological diseases and persisting 3 to 12 years after the diagnosis of infectious mononucleosis. To test the hypothesis if mononucleosis or asymptomatic Epstein-Barr virus seroconversion may predispose to hypersomnia we made systematic serological testing in a consecutive patients with diagnosed IH.

## Methods

### Population

Over a 5-year period, 20 patients aged 26.1 ± 9 years (range 18–56 years) coming to our sleep clinics for hypersomnia and daytime sleepiness were examined by a face-to-face clinical interview, sleep diary, 2-weeks actimetry and 24 h polysomnography. Among this sample, 14 patients meet the criteria for idiopathic hypersomnia, that is complaints of excessive daytime sleepiness for >1 year; – a night-time length >10 h for 3 weeks before polysomnography assessed by sleep diary; – the absence of narcolepsy-cataplexy, sleep irregularities and sleep deprivation symptoms; – lack of neurological or psychiatric diseases and respiratory and/or motor sleep disorders. Ten patients free of medication meet all the inclusion criteria and accepted blood samples including analysis for mononucleosis infection.

The local Ethics Committee approved the study. All patients gave their written inform consent prior to participation in the study.

## Clinical, microbiological, and instrumental assessment

### Clinical assessment

Clinical evaluation for patients was assessed by a standard clinical interview, which included history of the actual symptoms, the age of onset of the symptoms, their familiarity, the usual sleep-wake rhythm during working period and week-end period, the difficulties to waking up and the methods used to wake up and the number, type (refreshing/unrefreshing), and duration of naps. HAD scale as well as Beck questionnaire allowed us to exclude underlying depressive pathology or disease anxiety. Symptomatology suggestive of dysautonomia was questioned. The presence of orthostatic hypotension with syncope was found in 4 patients and the presence of chronic non-migraine headache in 2 others. There was no complaint about thermogenesis in this population.

The body mass index (BMI), calculated as weight/height squared (kg/m^2^), was measured and subjective sleepiness was evaluated using the Epworth Sleepiness Scale (ESS) questionnaire with an ESS>10 defining subjective sleepiness.

### Serological and blood sample analysis

Blood sample analysis include screening for diabetes, hypo-vitaminosis, anemia, iron deficiency, and serology to assess the presence of Epstein-Barr virus (EBV) infection. The serological analyses were made in the same laboratory, using the immunofluorescence assays (11) and including the three common serological tests to diagnose EBV: 1. Viral Capside Antigen (VCA) IgG, VCA IgM, and EBV nuclear antigen (EBNA). The laboratory serological reference values were respectively: VCA IgG: <200 U/ml, VCA IgM <500 U/ml, Virus Epstein Barr EBNA <800 U/ml). These tests make it possible to define the status of the infection. The presence of VCA IgG and VCA IgM without EBNA indicate acute infection whereas the presence of VCA IgG and EBNA in the absence of VCA IgM is typical of past infection (12). HLA-DRB1^*^1501-DQB1^*^0602 haplotype was absent in all patients.

### At-home 24-h polysomnography recording

Actimetric recording prior to polysomnography eliminated sleep phase shift phenomena, sleep restriction. The estimated average sleep time was 605 ± 40 min by this Actigraphic method.

In all subjects, a 24 h ambulatory *ad libitum* polysomnography was performed including conventional measures: four conventional electroencephalographic tracings, right and left electrooculogram, chin and bilateral anterior tibialis electromyograms, electrocardiogram (ECG), nasal pressure, respiratory effort, body position, and oxygen saturation, the latter is measured by pulse oximetry. All signals were recorded at 256 Hz. During the recording, the subjects were allowed to perform usual activities during daytime period and to sleep whenever they wanted according to their usual bedtime and waking up schedule reported in the sleep diary. Sleep stages were visually scored in 30-s epochs according to standard AASM criteria (13).

## Results

Clinical, polysomnographic and serological data are reported in Table 1. Ten patients aged 21.4 ± 2.6 years, nine females and 1 men, with a mean BMI of 21.4 ± 2.9 kg/m^2^ were examined. The mean age of symptoms onset was 18.9 ± 5.6 years. and their mean Epworth sleepiness score was 11.3 ± 5.4. All patients had cerebral magnetic resonance imaging that exclude the presence of cerebral lesion. None of the patients had metabolic alterations, including anemia, inflammatory indices and low levels of iron and ferritin. None of the patients reported infection prior the onset of sleepiness and hypersomnia and familiarity for hypersomnia.

**Table 1:** Clinical, biological, and sleep data for all subjects (mean ± SD).

Age (years)	22.2 ± 1.9
Age of onset (years)	18.1 ± 1.5
Body mass index (kg/m^2^)	20.8 ± 1.7
Epworth sleepiness scale	11.2 ± 0.93
Total sleep time (min)	570 ± 47.5
Awakenings *(n)*	14.8 ± 1.2
Sleep efficiency (%)	93.3 ± 7.8
Sleep latency (min)	16.7 ± 28.5
REM latency (min)	123.5 ± 61.8
Stage 1%	6.0 ± 0.5
Stage 2%	52.2 ± 4.3
Slow wave sleep %	21.0 ± 1.8
Rapid-eye-movement sleep%	19.8 ± 5.9
IgG (U/ml)	1010.74 ± 84.2
IgM (U/mL)	74.85 ± 0.6.2
EBNA /ml	1804.3 ± 150.4

The patients slept well with a total sleep time of about 10 h and a sleep efficiency of 94% indicating good sleep quality. None of the patients had respiratory events and periodic leg movements. In the bottom part of the Table 1 are reported the mean values ± SD of the serological results. All patients had high levels of VCA IgG and EBNA with low level of VCA IgM, indicating past infection.

## Discussion

To the best of our knowledge, the present study is the first to evaluate the role of Epstein Barr infection in patients with primary IH and without neurological disorders. Although none of the patients reported at the beginning of the disease some infection, we found the presence of the classical serological markers of a prior mononucleosis infection. We know that mononucleosis and Epstein-Barr infection are often asymptomatic and the clinical symptoms and their duration are variable. As reported by Lambore and coworkers in student community (14), patients with previous mononucleosis differ from those having other infections for greater and significant tiredness, sleepiness, and depression persisting after 1 year. Overall, these symptoms overlap with “Sickness Syndrome” (15) that is an adaptative host response to infectious in that in addition to fever and hypoferremia fatigue and hypersomnia are present, the last symptoms overlapping those described by HI patients.

Our study has several limitations. The first one is represented by the fact that not all patients met the ICSD-3 criteria for IH definition. In particular, 4 of them did not have a TST >660 min on 24 h polysomnography. However, the clinical picture was obvious (as well as the actimetry) and the subsequent follow-up of these 4 patients confirmed the presence of chronic hypersomnia. The lack of systematic realization of multiple sleep latency test (MSLT) is also a diagnostic limit (to eliminate type 2 narcolepsy) even if we know the limits of this test in sensitivity and specificity (16) in such a hypersomnia.

The second one is the absence of a control group in our study. However, the seroconversion rate for the Epstein-Bar virus is fairly well described in the literature and never reaches 100% (as we observed in our IH population) in all the cohorts of students assessed at college entry in the United States (17).

Our findings supports the hypothesis (18) of a possible role of autoinflammatory processes and immune dysregulation mechanism in young adults with IH.

## Author contributions

ES: patients examination, scoring the PSG, design of the study, writing the manuscript; DH: writing and editing the manuscript; FR: design of the study, writing the manuscript (more particularly: table, methods).

### Conflict of interest statement

The authors declare that the research was conducted in the absence of any commercial or financial relationships that could be construed as a potential conflict of interest.

## References

[1] Anderson KN Pilsworth S Sharples S Sharples LD Smith IE Sheerson JM. Idiopathic hypersomnia: a study of 77 cases. Sleep (2007) 30:1274–81. 10.1093/sleep/30.10.1274 17969461 PMC2266276

[2] Billiard M Dauvilliers Y Idiopathic hypersomnia. Sleep Med Rev. (2001) 5:349–58. 10.1053/smrv.2001.0168 12530998

[3] Vernet C Leu-Semenscu S Buzare MA Arnulf I. Subjective symptoms in idiopathic hypersomnia: beyond excessive sleepiness. J Sleep Res. (2010) 19:525–34. 10.1111/j.1365-2869.2010.00824.x 20408941

[4] Ali M Auger RR Slocumb NL Morgenthaler TI. Idiopathic hypersomnia: clinical features and response to treatment. J Clin Sleep Med. (2009) 5:562–8. 20465024 PMC2792973

[5] Nishino S Kanbayashi T. Symptomatic narcolepsy, cataplexy and hypersomnia, and their implications in the hypothalamic hypocretin/orexin system. Sleep Med Rev. (2005) 9:269–310. 10.1016/j.smrv.2005.03.004 16006155

[6] Huang ZL Mochizuki T Qu WM Hong ZY Watanabe T Urade Y. Altered sleep-wake characteristics and lack of arousal response to H3 receptor antagonist in histamine H1 receptor knockout mice. Prog Natl Acad Sci USA. (2006) 103:4687–92. 10.1073/pnas.0600451103 16537376 PMC1450232

[7] Lin JS. Brain structures and mechanisms involved in the cortical activation and wakefulness, with emphasis on the posterior hypothalamus and histaminergic neurons. Sleep Med Rev. (2000) 4:471–503. 10.1053/smrv.2000.0116 17210278

[8] Roehrs TA Tietz EI Zorick FJ Roth T. Daytime sleepiness and antihistamines. Sleep (1984) 7:137–41. 10.1093/sleep/7.2.137 6146180

[9] Kanbayashi T Kodama T Kondo H Satoh S Inoue Y Chiba S. CSF histamine contents in narcolepsy, idiopathic hypersomnia and obstructive sleep apnea. Sleep (2009) 32:181–7. 10.1093/sleep/32.2.181 19238805 PMC2635582

[10] Guilleminault C Mondini S. Mononucleosis and chronic daytime sleepiness. A long-term follow-up study. Arch Intern Med. (1986) 146:1333–5. 10.1001/archinte.1986.00360190107014 3718130

[11] Corrales I Giménez E Navarro D. Evaluation of the architect Epstein-Barr-Virus (EBV) viral capsid antigen (VCA) IgG, IgM, and EBV nuclear antigen 1IgG chemoluminiscent immunoassays for detection of EBV antibodies and categorization of EBV infection status using immunofluorescence assays as the reference method. Clin Vacc Immunol. (2014) 21:684–8. 10.1128/CVI.00104-14 24623623 PMC4018887

[12] De Pascale M Clerici P Serological diagnosis of Epstein-Barr virus infection: problems and solutions. World J Virol. (2012) 1:31–43 10.5501/wjv.v1.i1.31 24175209 PMC3782265

[13] Iber C Ancoli-Israel S Chesson AL Quan SF The AASM Manual for the Scoring of Sleep and Associated Events: Rules, Terminology, and Technical Specifications, 1st Edn. Westchester, IL: American Academy of Sleep Medicine (2007).

[14] Lambore S McSherry J Krauss AS. Acute and chronic symptoms of mononucleosis. Am Pract. (1991) 33:33–7. 2056288

[15] Shakar K Shakar G Why do feel sick when infected - can altruism plays a role? PLoS Biol. (2015) 13:e1002276 10.1371/journal.pbio.1002276 26474156 PMC4608734

[16] Billard M Sonka K Idiopathic hypersomnia. Sleep Med Rev. (2016) 29:23–33. 10.1016/j.smrv.2015.08.007 26599679

[17] Crawford DH Macsween KF Higgins CD Thomas R McAulay K Williams H. A cohort study among university students: identification of risk factors for epstein-barr virus seroconversion and infectious mononucleosis. Clin Infect Dis. (2006) 43:276–82. 10.1086/505400 16804839

[18] Barateau L Lopez R Arnulf I Lecendreux M Franco P Drouot X Comorbidity between central disorders of hypersomnolence and immune-based disorders. Neurology (2017) 88:93–100. 10.1212/WNL.0000000000003432 27864522

